# Electronic health databases and surveys for community-specific health promotion, prevention, and interventions: the Bangkoknoi Model Project (BANMOP)

**DOI:** 10.1186/s12913-025-12837-z

**Published:** 2025-05-10

**Authors:** Naris Kitnarong, Weerasak Muangpaisan, Pennapa Kaweewongprasert, Sichon Luerithiphong, Suporn Apinuntavech, Boonying Manaboriboon, Pattarawalai Talungchit, Siwaporn Chainuvati, Varalak Srinonprasert, Sukree Kade, Kantamas Supamanee, Prasert Assantachai, Prasit Watanapa

**Affiliations:** 1 Department of Ophthalmology, Faculty of Medicine Siriraj Hospital, Mahidol University, 2 Prannok Road, Bangkok, 10700 Thailand; 2 Department of Preventive and Social Medicine, Faculty of Medicine Siriraj Hospital, Mahidol University, Bangkok, Thailand; 3 Department of Psychiatry, Faculty of Medicine Siriraj Hospital, Mahidol University, Bangkok, Thailand; 4 Department of Pediatrics, Faculty of Medicine Siriraj Hospital, Mahidol University, Bangkok, Thailand; 5 Department of Obstetrics and Gynecology, Faculty of Medicine Siriraj Hospital, Mahidol University, Bangkok, Thailand; 6 Department of Internal Medicine, Faculty of Medicine Siriraj Hospital, Mahidol University, Bangkok, Thailand; 7 Siriraj Applied Thai Traditional Medicine, Faculty of Medicine Siriraj Hospital, Mahidol University, Bangkok, Thailand; 8 Division of corporate social responsibility, Faculty of Medicine Siriraj Hospital, Mahidol University, Bangkok, Thailand; 9 Department of Surgery, Faculty of Medicine Siriraj Hospital, Mahidol University, Bangkok, Thailand

**Keywords:** Electronic health database, Health promotion, Elderly, Community health

## Abstract

The Bangkoknoi Model Project (BANMOP)is a prospective cohort project and is guided by context-specific health databases to promote the sustainable health and well-being of people living in the Bangkoknoi district. The BANMOP emphasized community engagement via electronic databases. Data were collected from households in the Bangkoknoi district during November 2017 to January 2019. All voluntary participants were included and data were collected via mobile application, a web-based platform, and by face-to-face interviews with well-trained volunteers who were mainly health professionals. Data were categorized by age groups: 0–5, 6–14, 15–21, 22–59, > 60 years old, included both individuals and families in five categories: health, environment & disaster, economics, social, and safety. A total of 22,510 participants from 42 Bangkoknoi communities represented 17% of the total population of the Bangkoknoi district. 44% of the participants reported having physical health problems. Noncommunicable diseases were dominated by physical health problems as age progressed. Overall, hypertension was the most prevalent health problem at 10.8% followed by allergic diseases (7.6%), diabetes mellitus (5.2%), and dyslipidemia (3.1%).Anxiety was the most common mental health disorder, and gender income disparity was an important issue. Although, 91.6% of participants satisfied with their home environment, but the crowded, cluttered, urban environment caused their dissatisfaction. 42% reported problems related to reproductive health such as teen pregnancy (16.7%), family planning and marriage guidance (12.7%), premature sexual transmitted diseases (9.0%) and infertility (3.8%). Subsequently, 20 sub-projects were established to promote the sustainable health and well-being of people in the Bangkoknoi district. BANMOP can provide not only context-specific health information, but also health-related issues that are often neglected. The community engagement and local data supplemented to NHES are needed for sustainable community-specific health policies and interventions. The information that newly discovered from BANMOP such as allergy in childhood and mental health problem needs to be further explored.

**Trial registration** ClinicalTrials.gov identifier: NCT06583694 (Retrospectively registered on September 4, 2024).

## Introduction

### Thailand’s health care system and its challenges

Thailand has three major health care schemes that cover approximately 95.5% of the Thai population including the civil service welfare, the social security scheme and the universal health coverage scheme [1, 2]. Currently, the public health system is managed by the Ministry of Public Health in conjunction with other health-related agencies, such as the National Health Security Office (NHSO). Thailand’s health system faces major challenges from aging of the population and rapid urbanization [3]. The adaptation of existing living spaces should be considered to allow older people to live with other generations, a concept known as “aging in place” [4, 5]. Healthcare organizations should proactively work to make cities healthier and not simply wait for people to be injured or ill caused by their urbanized environment [6]. Moreover, healthcare should not be the sole responsibility of the government, nor should we rely solely on healthcare organizations to deliver such results. It is the responsibility of every organization, community and individual to help improve the health and well-being of people in their communities [7, 8]. 

Since 1991, the Thai National Health Examination Survey (NHES) has been conducted every five years by the Ministry of Public Health (MOPH). The NHES focuses on NCD and associated risk factors. Due to the 5-year interval between surveys, the NHES is unable to respond to dynamic changes to inform policy development. Second, the NHES takes a broad national perspective, combining data from different regions in rural and urban settings. Third, the NHES does not benefit from community engagement nor does it provide prompt feedback to communities. Finally, the NHES is a cross-sectional study and therefore the incidence of disease, many health outcomes, and causal relationships cannot be established.

The Bangkoknoi Model Project (BANMOP) was established in 2015 by the Faculty of Medicine of Siriraj Hospital, Mahidol University, in partnership with the Thai Health Promotion Foundation [8]. The BANMOP aims to promote the sustainable health and well-being of people living in the Bangkoknoi district and is guided by context-specific health databases and with the collaboration of community stakeholders. The BANMOP also aims to provide data to complement NHES [9]. Upon completion, BANMOP will be able to provide not only context-specific health information, but also health-related information (environment & disaster, economics, social, and safety) to help design healthcare policies and interventions. This is the first report of BANMOP presenting the results of the electronic health databases gathered during the second phase (cross-sectional study) of BANMOP between November 2017 to January 2019.

## Materials and methods

**Fig. 1:**
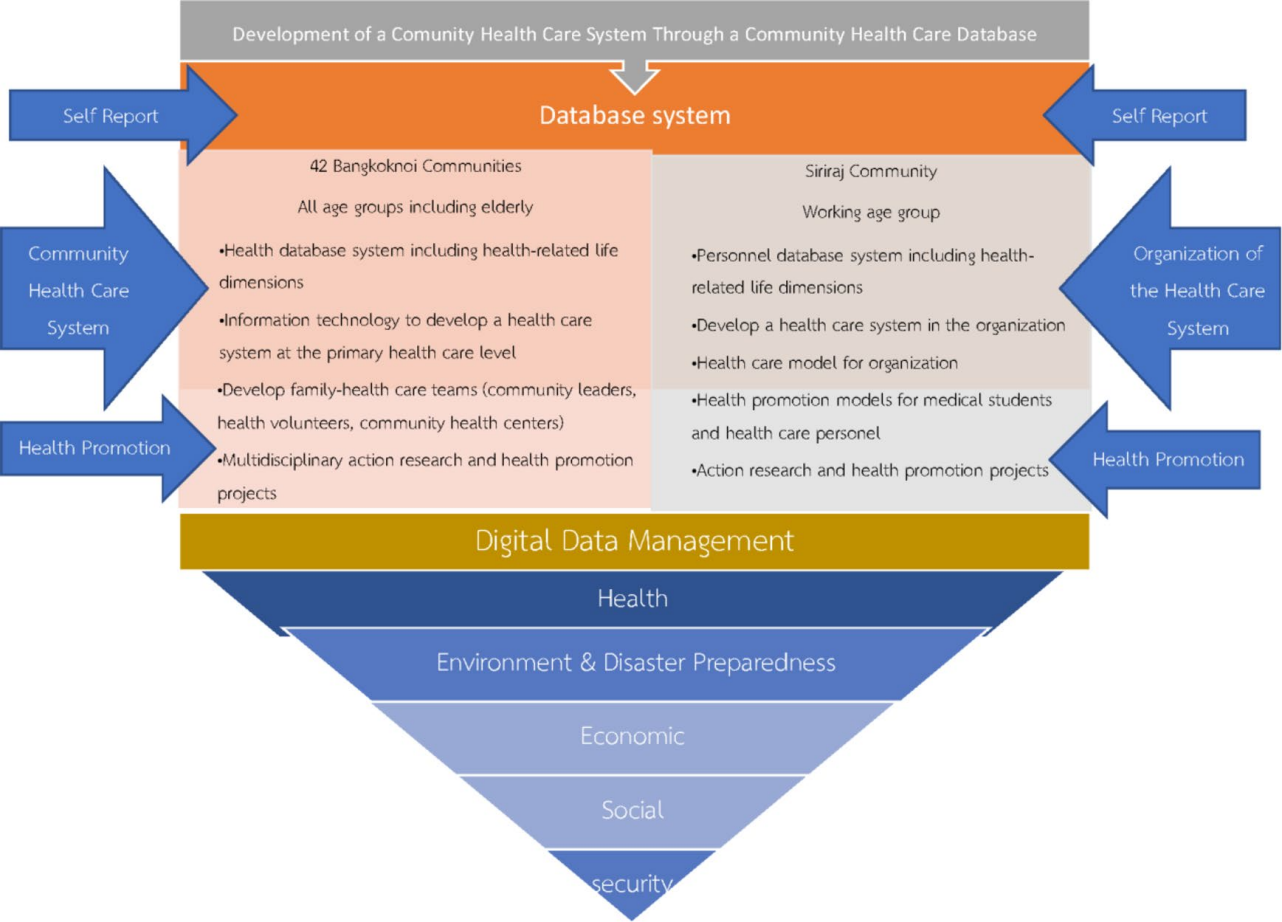
Bangkoknoi Model Project conceptual framework

The BANMOP was divided into three phases over 4.5 years starting from 1 October 2015 to 31 March 2020. The first phase (October 2015 to October 2017) was designed to develop questionnaires and databases for health information, and the networks and management systems for healthcare were created. The second phase (November 2017 to January 2019) focused on data collection and worked with local community leaders in the Bangkoknoi district and partnering organizations. The third phase (February 2019 to March 2020) focused on data analysis and the development of projects to promote positive health outcomes in the Bangkoknoi district. Beside the Faculty of Medicine of Siriraj Hospital and the Thai Health Promotion, the other major stakeholders include 42 local communities, the Royal Thai Navy, 22 primary schools, 6 secondary schools, the Bangkoknoi District Bureau and the Office of the Basic Education Commission. The Bangkoknoi district is one of 50 districts in Bangkok, the capital of Thailand. The conceptual framework of BANMOP is demonstrated in Fig. 1.

### Development of a survey instrument

Survey questionnaires were developed through a collaboration of ten departments of the Faculty of Medicine of Siriraj Hospital, Mahidol University. The Thai language questionnaires were developed as a mobile device application and web-based platforms. There were two types of questionnaires, household and individuals (Table 1). For individual questionnaires, there were 5 age-specific questionnaires (0–5, 6–14, 15–21, 22–59, > 60 years old). The content of the questionnaires covered health (80%), environment & disaster (6%), economics (6%), social (4%) and safety (4%). The respondents were classified by age group: 0–5, 6–14, 15–21, 22–59, > 60 years old with different questions for different age groups. A member of each household completed a family questionnaire. For the Siriraj hospital personel, an additional questionnaire was completed to measure happiness in working in the hospital (happinometer). After the questionnaire was developed, community representatives were invited to provide feedback and suggestions to adapt the questionnaire to the local context.

**Table 1:** The Bangkoknoi Model Project data elements

Individual questionnaire	
Demographic Data • Age • Gender • Education attainment • Order of child among siblings • Socioeconomic level • Number of caregivers Economics • Incomes • Employment (full-time/part-time) • Income expense and saving records • State welfare	Health-related information • History of maternal antenatal care and breastfeeding • Vaccination/childhood development • Underlying diseases/disabilities • Medications • Dental health and care history • Health Insurances/coverage/scheme • Addictive substance used • Reproductive health-related history • Mental health status and care • Physical activities and exercises • History of traditional medicine healthcare
Household questionnaire (performed by one member of each household)	
Environment & disaster • Housing and problems • Water & Sanitation • Waste & Garbage management • Insects/vectors • Relationship of people in the family • Family communication • Family together time Safety • Housing safety-related issues • Lighting, Pathway • Crime prevention • Fire prevention • Knowledge of how to access the emergency contact number	Social • Daily lifestyle • Assigned position/duty in the community • Relationship of people in the community • Neighborhood hospitalities • Community support • Parental status and family relationship • School problems • Time spent on the computer • A place for physical activities and exercises • Volunteer and community activities • Religion and related activities • Community reproductive service • Community wisdom
For Siriraj Community	
For medical students • Similar to questionnaires for 42 communities, but including educational and medical school-related questions.	For hospital staff • Similar to the questionnaire for 42 communities, but questions related to hospital work and additional questions based on modified Mahidol happinometry.

### Data collection team management

#### (Recruiting, training, and Building BANMOP community network management team)

The interviewers were recruited from the Siriraj Hospital Faculty of Medicine (health professionals and administrative officers) and community volunteers. During home visits, the interviewers also observed the structure and environment of the housing. When participants reported having an underlying medical condition, interviewers checked for prescribed medications to confirm the diagnosis. The BANMOP-communities network management team was established to oversee this process. This team consisted of researchers and coordinators in the communities (one coordinator per 4–5 communities), and teacher coordinators in school networks (a coordinator for two secondary schools & a coordinator for each primary school) [10]. 

#### Visitation processes [10]

First, a community coordination meeting was organized with the heads of local communities. Then an orientation was held for volunteers to analyze the communities. Finally, the home visiting process to gather data was implemented from November 2017 to January 2019. This involved first meeting with mentors in the designated zones to receive their advice, walking into the community as a team, recording the data, and solving immediate problems in the communities as they arise.

### Data collection

#### Study participants

The data resource area boundaries are (North) Bang Phlat district, (East) Chao Phraya River, (West) Chak Phra Canal and Taling Chan district, (South) Morn Canal, and Bangkok Yai district. The total population of Bangkoknoi district was 132,372 in 48,771 households.

#### Case definitions

Individuals who met the following criteria were included in the survey.

1. Individuals registered with the Bangkoknoi district and currently reside there.
2. Individuals who are not registered, but currently reside in the Bangkoknoi district.
3. Individuals registered in the Bangkoknoi district, but who currently reside outside the district.
4. Individuals, Thai and non-Thai citizens, who work continuously in the Bangkoknoi district for at least 30 days.
5. Individuals who study in schools or universities in the Bangkoknoi district continuously for at least 30 days.

The minimum sample size was calculated using Cochran’s 1963 sample size formula [11] using the large sample normal approximation for a single proportion to achieve a two-sided 95% confidence level with extended 0.006 from the observed proportion for an expected proportion of 0.257 [12, 13]. The minimum sample needed was 20,378. Estimating incomplete data and the missing cases of 10%, we expanded the predicted sample size to 22,414. The convenient sampling method was used in this study, which included all willing participants who were willing to participate face to face or through self-reporting via a mobile application or web-based platform (www.bangkoknoimodel.com).

Individuals who were not willing to participate or answer the questionnaires were excluded. All participants provided verbal and/or electronic informed consent before participation. ‘Volunteers’ were defined as Siriraj healthcare professionals who were trained to collect data.

### Data analysis

Data were collected by mobile application or web-based platform, and by face-to-face interviews with trained volunteers, mainly health professionals. The Global Positioning System (GPS) was used to locate the home of each participant. Data analysis and visualization were performed on the Kibana Platform System provided by HUAJAI IT Co., LTD.

### Ethical clearance & funding sources

BANMOP received ethical approval from the Institutional Review Board, Siriraj Hospital Faculty of Medicine, Mahidol University (SIRB) in accordance with the tenets of the Declaration of Helsinki on December 27, 2016. The SIRB number is AA59000801, and EC No.SI 820/2016. The project was funded by the Thai Health Promotion Foundation (grant number 58-00-232) and the Siriraj Hospital Faculty of Medicine of Mahidol University.

## Results

A total of 30,335 people participated in the BANMOP survey, which includes 7,825 staff from Siriraj Hospital. and 22,510 residents of Bangkoknoi communities. The total of 22,510 enrolled participants from 42 Bangkoknoi communities accounted for 17% of the population of the Bangkoknoi district who responded on individual questionnaires. When stratified by sex, 13,074 (63.68%) were women and 9,436 (36.32%) were men. When stratified by age group, there were 1,171 (5.20%) of 0–5 year-olds, 5,857 (26.02%) of 6–14 year-olds, 4,257 (18.91%) of 15–21 year-olds, 7,382 (32.8%) of 22–59 year-olds, and 3,842 (17.07%) of over 60 year-olds. Nearly four-fifths (77.88%) had some form of healthcare scheme such as the universal health coverage scheme or the social security scheme, and one-fifth (20.19%) did not know what health care scheme they were eligible for. There were 10,072 household representatives who responded to the household questionnaires.

### Health related issues

#### Physical health

A total of 10,043 (44.62%) of 22,510 participants reported having physical health problems. Health problems increase as age advances. Children under 5 years of age had the lowest health problems at 19.96% compared to 24.73% (6–14 year-olds), 26.76% (15–21 year-olds), and 42.71% (22–59 year-olds), while 79.95% of those over 60 years of age had health problems.

In general, hypertension was the most prevalent health problem at 10.8% followed by allergic diseases (7.6%), diabetes mellitus (5.2%), dyslipidemia (3.1%), and bone and joint diseases (2.2%).

**Fig. 2:**
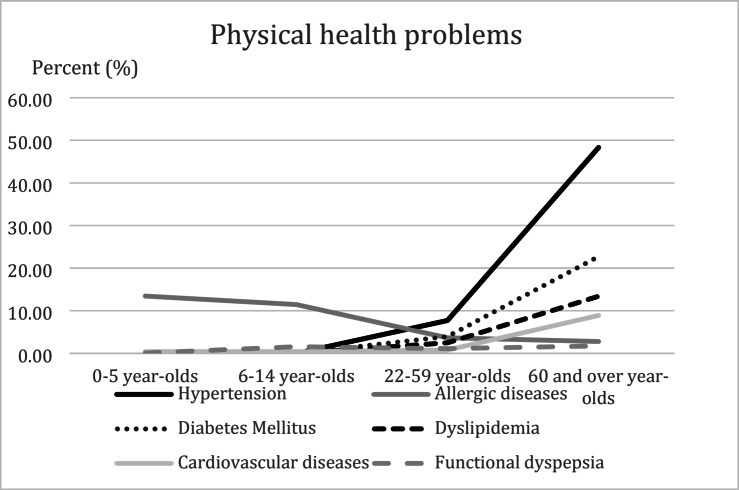
Percentage of each physical health problem in each age group

We found that the prevalence of hypertension increases with age and reached the highest prevalence of 48.3% in the age group of over 60 years. Other NCD, including diabetes mellitus and dyslipidemia, also had similar trends. On the contrary, allergic-related diseases and functional dyspepsia reached a peak prevalence at 15–21 years of age and then began to decrease. The percentage trends for each physical health problem in each age group are shown in Fig. 2.

For the 0–5 age group, the top 3 most prevalent health problems included allergic-related diseases, respiratory diseases, and hematologic/anemia problems. For the 6–14 and 15–21 age group, the 3 health problems that were the most prevalent were allergy-related diseases, functional dyspepsia, and hematologic/anemia diseases. For people in the working period (22–59 age-group), the 5 most prevalent health problems were hypertension, diabetes, allergy-related diseases, dyslipidemia, and cancer. Finally, the 60 year and over age group had hypertension, diabetes, dyslipidemia, bone/joint problems, and cardiovascular diseases as the top 5 most common health problems. The demographic characteristics and physical health problems classified by age groups are demonstrated in Table 2.

**Table 2:** Demographic data and physical health problems categorized by age-groups

	Numbers total 22,510		0–5 1,171 (5.20%)		6–14 5,857 (26.02%)		15–21 4,257 (18.91%)		22–59 7,383 (32.80%)		60 and over 3,842 (17.07%)	
	persons	%	persons	%	persons	%	persons	%	persons	%	persons	%
Gender												
Male	9,436	41.92	596	50.86	2,398	42.65	1,315	30.88	2,167	29.35	1,266	32.94
Female	13,074	58.08	575	49.02	3,459	57.31	2,942	69.00	5,216	70.54	882	67.01
Hypertension	2,432	10.80	0	0	0	0	6	0.14	570	7.72	1,856	48.31
Allergic diseases	1,703	7.57	110	13.45	671	11.46	545	12.80	270	3.66	107	2.79
Diabetes Mellitus	1,174	5.22	1	0.09	0	0	8	0.19	292	3.96	873	22.72
Dyslipidemia	700	3.11	0	0	0	0	1	0.02	184	2.49	515	13.40
Bone and joint diseases	494	2.19	0	0	7	0.12	24	0.56	93	1.26	368	9.58
Cancer	454	2.02	2	0.17	21	0.36	65	1.53	184	2.49	182	4.74
Cardiovascular diseases	442	1.96	5	0.43	24	0.41	13	0.31	58	0.79	342	8.90
Peptic ulcer	406	1.80	1	0.09	92	1.57	163	3.83	83	1.12	67	1.74
Cataract, glaucoma, and pterygium	379	1.68	0	0	0	0	1	0.02	48	0.65	330	8.59
Renal diseases	287	1.27	7	0.60	28	0.48	39	0.92	84	1.14	129	3.36
Hematologic/Anemia	265	1.17	8	0.68	63	1.08	103	2.42	49	0.66	42	1.09
Paresis/paralysis	245	1.09	3	0.26	16	0.27	31	0.73	78	1.06	117	3.05
Thyroid diseases	178	0.79	2	0.17	9	0.15	16	0.38	77	1.04	74	1.93
Respiratory diseases	141	0.63	13	1.11	43	0.73	16	0.38	27	0.37	42	1.09
Dermatological diseases	133	0.59	5	0.43	36	0.61	27	0.63	38	0.51	27	0.70
Obesity	126	0.56	1	0.09	41	0.70	29	0.68	35	0.47	20	0.52
Intestinal diseases	112	0.50	4	0.34	17	0.29	31	0.73	20	0.27	40	1.04
Cerebrovascular diseases	95	0.42	0	0	0	0	1	0.02	19	0.26	75	1.95
Musculoskeletal diseases	90	0.40	1	0.09	2	0.03	4	0.09	32	0.43	51	1.33
Neurologic diseases	53	0.24	0	0	0	0	5	0.12	16	0.22	32	0.83
Epilepsy	46	0.20	5	0.43	11	0.19	6	0.14	17	0.23	7	0.18
Tuberculosis	29	0.13	0	0	0	0	2	0.05	10	0.14	17	0.44
Autistic	19	0.08	2	0.17	9	0.15	2	0.05	5	0.07	1	0.03
Psoriasis	18	0.08	0	0	0	0	2	0.05	12	0.16	4	0.10
Sexually transmitted diseases	4	0.02	0	0	0	0	1	0.02	1	0.01	2	0.05

#### Mental health

**Fig. 3:**
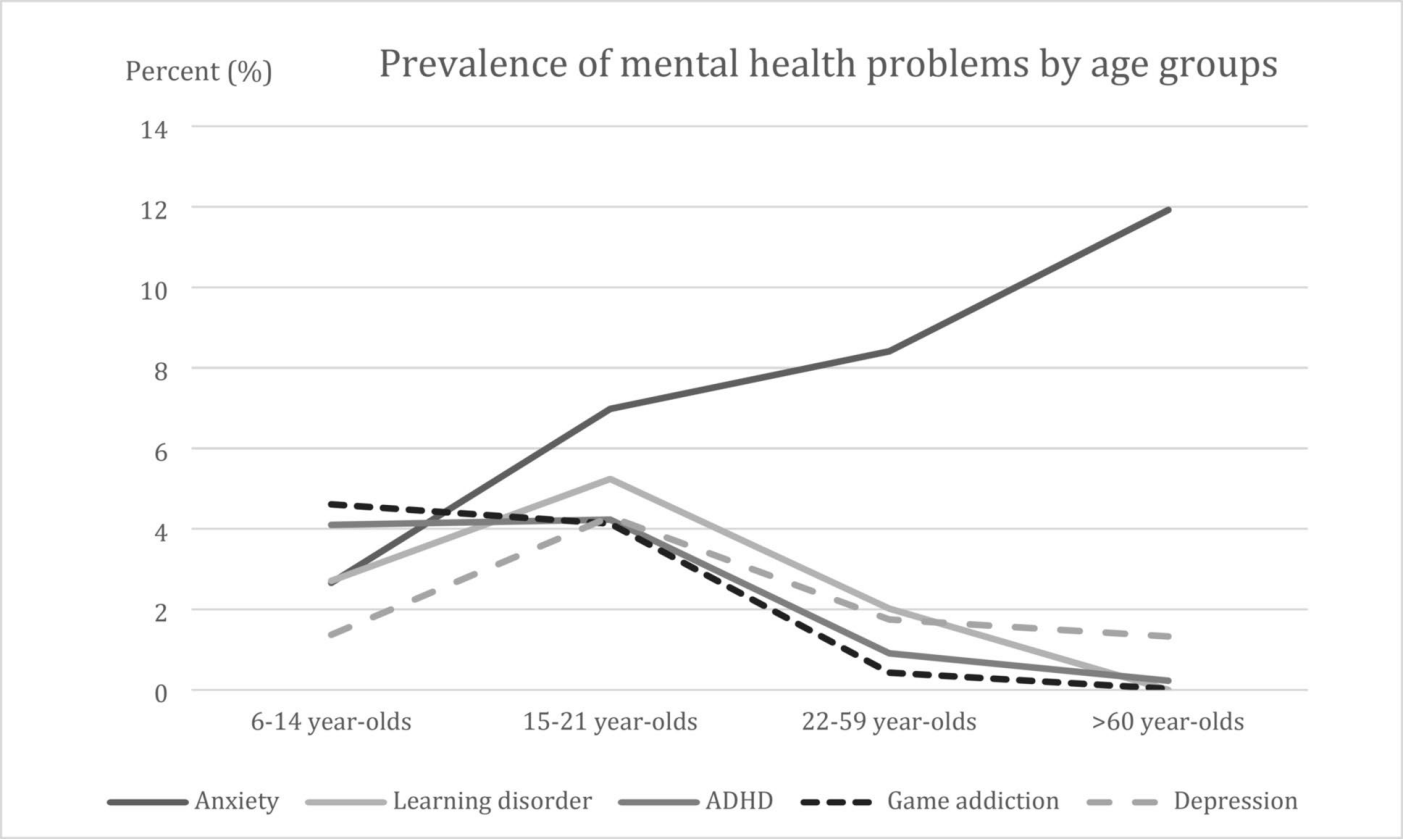
Prevalence (percentage) of mental health problems by age group

A total of 2,980 participants (13.2%) reported mental health problems. The prevalence of mental health problems was present 12.9% (756 participants) in the age group 6–14 years, 15.4% (655 participants) in the age group 14–21 years, 12.0% (886 participants) in the age group 22–59 years, and the highest 17.8% (683 participants) in the age group 60 years and over. Anxiety disorder had the highest prevalence at 6.8%, followed by learning disorders (2.4%), attention-deficit/hyperactivity disorder (ADHD) (2.2%), game addiction (2.1%), and depression (1.97%). The distribution of mental health problems among age groups is demonstrated in Table 3. The prevalence of anxiety disorder increased with age and reached a peak of 11.9% in those 60 years and older. In contrast, ADHD and learning disorders reached a peak prevalence in the 15–21 age group, then decreased in the older age groups. The trends for each mental health problem are shown in Fig. 3.

**Table 3:** Distribution of mental health problems among age groups

Numbers (%)	Total 2,980 (13.24%)		6–14 756 (12.91%)		15–21 655 (15.38%)		22–59 886 (12.00%)		60 and older 683 (17.78%)	
	persons	%	persons	%	persons	%	persons	%	persons	%
Anxiety	1,532	6.81	156	2.66	297	6.98	621	8.41	458	11.92
Learning disorder	531	2.35	159	2.71	223	5.24	149	2.02	0	0.00
Attention-deficit/hyperactivity disorder	497	2.21	240	4.10	180	4.23	67	0.91	9	0.23
Game addiction	479	2.13	270	4.61	176	4.13	32	0.43	1	0.03
Depression	443	1.97	80	1.37	183	4.30	129	1.75	51	1.33
Schizophrenia	39	0.17	6	0.10	8	0.19	20	0.27	5	0.13
Drug abuse	15	0.07	5	0.09	9	0.21	1	0.01	0	0.00
Unspecified	76	0.34	22	0.38	19	0.45	19	0.26	16	0.42
Others	194	0.86	29	0.50	34	0.80	82	1.11	49	1.28

### Substance abused

We found that 95% of people who answered this question never tried any substances. Although 4.7% had tried substances which 83.9% started during the second decade of their lives; 11–15 years old (42.1%) and 16–20 years old (41.9%).

### Environment, disaster, social and safety

The large majority (91.6%) of the participants indicated that they were satisfied with their home environment. The causes of dissatisfaction were related to the crowded and cluttered urban environment. The main causes of dissatisfaction with the surrounding environment were sound and dust. 21% of households lived near rivers and canals, and 80.3% of households thought that lighting was adequate in the community. 79% of households reported having sitting toilets, while 21% used a squat toilet with a high platform that can increase the risk of accidents.

Almost all (98.7%) of the households had adequate water supply and 95.1% of the supply was tap water. Only 20.8% of households had some type of wastewater treatment, such as septic tank, grease trap, or household wastewater treatment system. The rest of the households discarded wastewater through the drainpipe system without treatment. About a quarter of households dispose of wastewater directly into the river or other natural water sources. Although 98.7% of households had their own garbage containers, only 54.4% separated waste for recycling purposes. Moreover, only 15.3% reused items and/or sold to recycling centers.

When household representatives were asked what social/community problems should be addressed, 42.20% suggested problems related to reproductive health and families such as teen pregnancy (16.72%), family planning and marriage guidance (12.74%), sexually transmitted diseases (8.96%), and infertility (3.78%). On the other hand, more than 31.2% of the households did not know which problems to recommend and 20.4% said that there were no problems. The majority addressed substance abuse (1.8%) and crime (0.4%).

Most participants (82.97%) felt that their communities were safe. Around two-thirds of households (66.63%) reported having some knowledge about fire safety plans, 33.37% did not have knowledge of fire safety.

### Financial issues

**Fig. 4:**
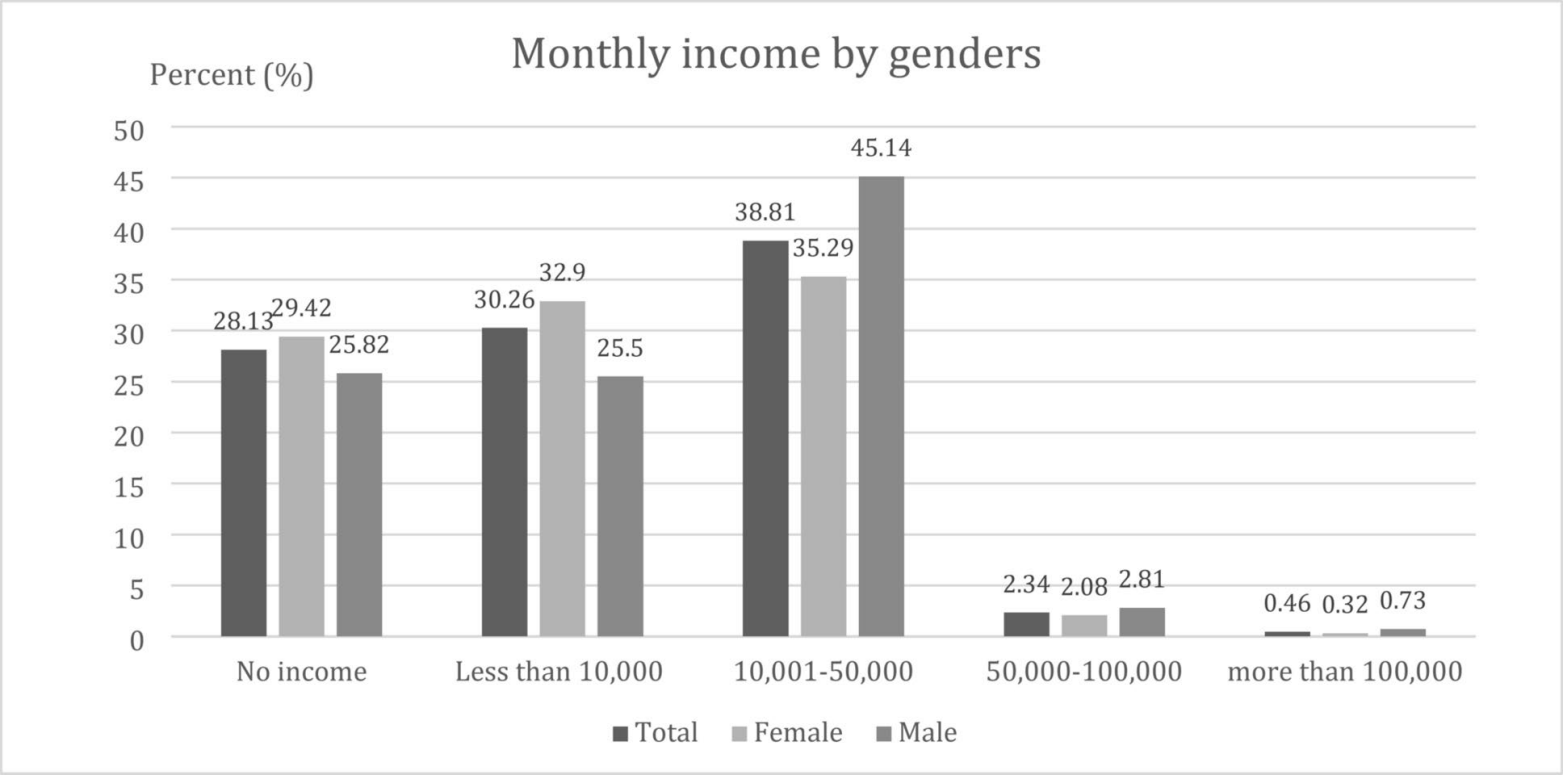
The distribution of individual monthly incomes by sex

Most (72.7%) households were single/nuclear families and 36.8% of households had at least one family member that needed special care, such as the elderly (50.9%) and children under 5 years of age (23.4%). 38% of the participants reported monthly individual income between 10,001 and 50,000 Thai baht (approximately 303-1,516 USD). We observed female-male income disparity as incomes increase, and we found that the male-to-female ratio was reversed at higher income levels. The distribution of monthly individual incomes by sex is demonstrated in Fig. 4.

**Fig. 5:**
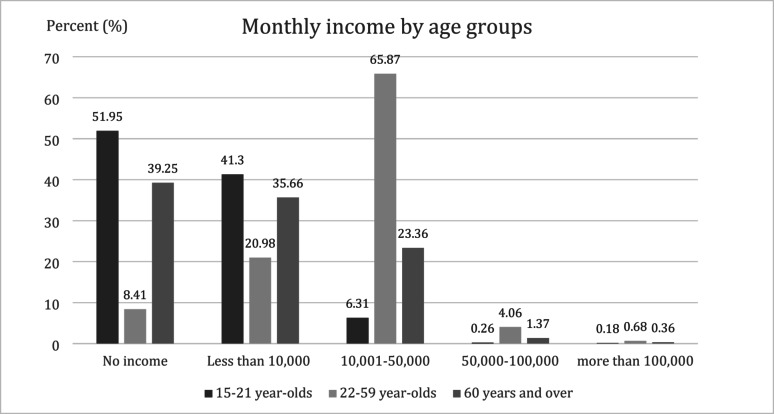
The levels of individual monthly income stratified by age groups

When stratified by age groups, most participants (65.9%) participants who were 15–21 years old had monthly income between 10,001 and 50,000 Thai baht. 39% of participants over 60 years of age (39.3%) had no income (excluding government elderly welfare), while 35.7% had an income of less than 10,000 Thai baht, and 23.4% had an income of 10,001–50,000 Thai baht. The proportion of participants who had income over 50,001 Thai baht ranged from 0.2 to 4.0%. Most of the participants in this income range report that they earn income from secondary or professional jobs. The levels of individual monthly income stratified by age groups are shown in Fig. 5.

**Fig. 6:**
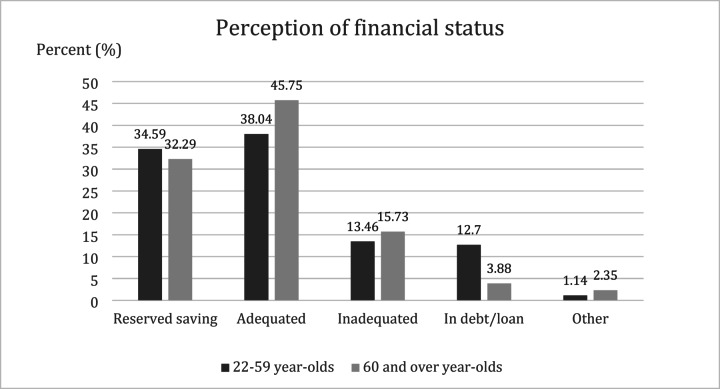
Perception of financial status stratified by age groups

Most of the participants (79.9%) indicated that they have adequate income with and without savings. Unlike monthly income, no gender disparity was observed. We found that most of the two age groups of 22 to 59 years and older than 60 years reported having adequate income with or without reserve savings. 12% of 22–59-year-olds and 3.88% of age groups over 60 years of age disclosed that they were in debt or had received a loan. In contrast to perception of financial status, most households (86.7%) have not recorded their income and expenses. The perception of financial status stratified by age groups is demonstrated in Fig. 6.

## Discussion

### Representativeness

**Fig. 7:**
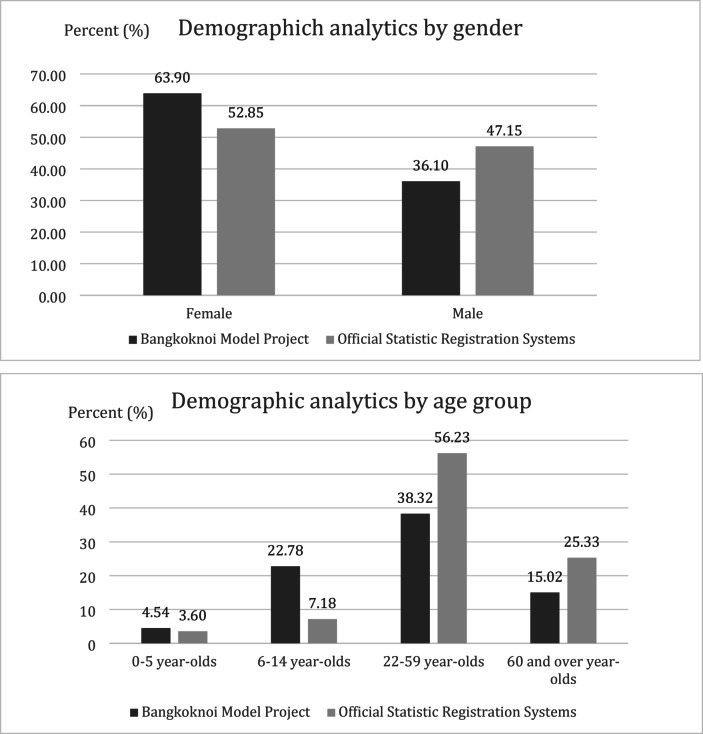
Comparison of sex and age-group distribution between participants in the Bangkoknoi model project and the household members of the official statistics registration system

The comparison of the sex and age distribution between the participants in the Bangkoknoi model project and the household members of the official statistics registration system are shown in Fig. 7. The participants were similar in age and sex to the data reported by the official statistics registration system. BANMOP has a higher female population (63.90%) compared to 52.85% in the official statistics registration system. Compared to the official statistics registration system, BANMOP has a slightly higher proportion of people in the age group 0–5,6–14,15–21 and over 60, but a lower proportion in the age group of 22–59. One possible explanation is that some people may have been working and not at home when the survey was conducted.

### Health related issues

Among the most common noncommunicable diseases (NCDs) in BANMOP, we discovered that the prevalence of these conditions rose with age and included hypertension (10.8%), diabetes mellitus (5.2%), and dyslipidemia (3.1%). Given the rise in NCD-related deaths over the past few decades, diabetes and hypertension have emerged as the two main NCDs and have become a significant global public health concern [14]. This finding holds true for a number of Asian nations [15–17]. Chronic obstructive pulmonary disease (COPD), heart disease, stroke, diabetes, and hypertension were the five main NCDs in China [15]. In a population-based cross-sectional study, Haidari et al. documented the prevalence of common NCDs in Lebanon, such as cancer (3.7%), diabetes mellitus (26.8%), cardiovascular disease (16.1%), asthma (7.1%), and hypertension (32.8%) [17]. Despite some variations in the most prevalent NCDs across nations, diabetes and hypertension consistently rank among the top three NCDs worldwide.

According to the fifth Thai National Health Examination Survey [NHES V] in 2014, the prevalence of hypertension in the population aged 15 years or older was 24.7% compared to 15.7% in BANMOP. The prevalence of hypertension in BANMOP in 22–59-year-olds is around 7.7% and 48.3% in those 60 and over years old. One possible explanation is that the percentage of hypertension in the age group of 22 to 59 years was low because 45% of hypertension are unaware according to NHES V.

The prevalence of allergic diseases, which were not frequently reported in earlier studies, was the second most prevalent NCD in BANMOP. Among those under 20, the prevalence of childhood and adolescent allergic diseases ranged from 11.5 to 13.5%. The population and location under study may have an impact on this particular BANMOP finding. The Bangkoknoi district is a Bangkok suburb that is presently becoming more urbanized. The local population is at risk of being exposed to a polluted environment, which leads to a high prevalence of diseases related to allergies. These questions need to be investigated further.

The prevalence of mental health problems in the BANMOP was slightly higher than in the Thai National Mental Health Survey in 2013. These differences might be caused by the use of different types of tools and age group stratification. Although the 2013 Thai National Mental Health Survey [18] used World Mental Health Composite International Diagnostic Interview version 3.0 (WMH-CIDI 3.0) that applies medical definitions and reports the percentage of the entire population in all age groups, BANMOP used a self-report system with stratification of age groups. Therefore, there was a possibility that the BANMOP survey captured population groups who felt that they have mental health problems, but did not yet match the disease criteria of WMH-CIDI 3.0 of NHES V.

### Socioeconomics

We compared the financial and economic data of BANMOP with similar data from the World Bank. Thailand’s 2020 GDP per capita (constant US$2010) was $6094.4 USD per year, or about 507.86 USD per month (16,251 baht per month [19]. In Bangkoknoi, 38.1% of the participants had a monthly income between 10,001 and 50,000 Thai baht (approximately 303-1,516 USD). Another BANMOP observation on the disparity between female and male incomes was in line with an existing study on the inequality of the gender wage gap in 2015 [20]. Additionally, the majority of households 86.68% did not record their income and expenses account, which can lead to future financial problems due to lack of planning. Poverty is one of the main contributors to poor health outcomes. Financial literacy should be one of the targets in intervention policies.

An outstanding point from the household survey was that almost half of the respondents (42.2%) identified that the social/community problems they wanted to solve were related to reproductive health and families such as pregnancy in adolescents (16.7%), family planning and marriage guidance (12.7%), sexually transmitted diseases (9.0%) and infertility (3.8%). This information is very helpful in designing health promotion and prevention programs that address the needs of the community.

### Dissemination and implications of the study findings

The BANMOP identified the similarities and differences that people living in Bangkoknoi district had compared to the National Survey. National surveys are a good starting point, but local data are needed to make community-specific interventions for sustained changes. Context-specific databases and strong partnerships with stakeholders are essential to ensure long-lasting success. A similarity in the data was physical health problems such as increased NCDs in the aging group. These will help the organization to emphasize an intervention that is similar to national policy. On the other hand, the information that differs or newly discovered from specific type of question beyond national survey needs to be further explored, such as allergy in childhood and adolescent and mental health problem that might be slightly higher.

To create ownership of the project and ensure sustained contributions, the BANMOP stakeholders met to discuss the study results and plan future projects. The stakeholders consisted of Siriraj Hospital Faculty of Medicine, Thai Health Organization, Bangkoknoi District Office, Mahidol University Faculty of Nursing, Royal Thai Navy, Office of the Basic Education Commission, Mahidol University International College, 42 communities in Bangkoknoi District and primary and secondary schools in Bangkoknoi District. After discussion and prioritization of stakeholders, 20 projects were established, including;

1. A workshop for CPR Volunteer Training Project in the Bangkoknoi Communities by the Department of Emergency Medicine.
2. Basic CPR classes for high school students by the Private Patient Division of the Department of Nursing, Faculty of Nursing, Mahidol University.
3. A research project by the Department of Emergency Medicine on “CPR Volunteers by Motorcycle Taxis” to help the people in the communities of Bangkoknoi to receive CPR before the Emergency Health Services arrive.
4. A research project on enhancing the physical health in the elderly by the Siriraj Health Policy Unit.
5. CSR with CBR (community-based rehabilitation) by the Department of Rehabilitation Medicine.
6. Bangkoknoi Place and People by Mahidol University International College.
7. Bangkoknoi Challenge for Well-being of the Elderly by the Siriraj Health Policy Unit and Siriraj Research Network.
8. A project on self-management of noncommunicable diseases in elderly patients by the Faculty of Nursing, Mahidol University.
9. A new generation of sex educators by the Department of Pediatrics, Faculty of Nursing, Mahidol University.
10. A project on the result of building motivation and self-control to avoid alcohol among the laborer population by the Department of Internal Medicine, Faculty of Nursing, Mahidol University.
11. Improving prehospital emergency care model for semi-urban area by the Department of Emergency Medicine.
12. Healthy Thai project 1: Community-driven air pollution alert system with Bangkoknoi model by the department of Internal Medicine.
13. Healthy Thai Project with Bangkoknoi Model: Fatless community by the Department of Internal Medicine.
14. Developing a management model of sexuality education and counselling in the secondary school, Bangkok Noi district, Bangkok by the Department of Pediatrics, Faculty of Nursing, Mahidol University.
15. The sustainable development of community-based rehabilitation program in urban Thailand by the Department of Rehabilitation Medicine.
16. Developmental surveillance and promotion manual (DSPM) by the Department of Pediatrics.
17. Development of health literacy for patients with hypertension in the community by the Department of Basic Nursing, Faculty of Nursing, Mahidol University.
18. Health coach for diabetes and hypertension prevention with a new normal in urban areas by the Siriraj Health Policy Unit and the Siriraj Research Network.
19. The Bangkoknoi model eye project: Prevalence of Glaucoma and Normative data of Ocular Biometry in Elderly Population in Suburban Area of Bangkok: The Bangkoknoi Model Project (BANMOP).
20. Health survey of monks residing in urban areas: The Bangkok Noi model.

BANMOP population data such as age, gender, incomes are available in a dashboard format at https://www.bangkoknoimodel.com/stat. In-depth data are available in Excel format in Thai and English at https://www.bangkoknoimodel.com/stat. To protect participant privacy, the researcher must submit a request form on the website and wait for approval from the BANMOP committee to access the database.

### Strengths and weaknesses

A significant strength of the BANMOP is the large number of people included in the survey. The most distinctive feature of BANMOP is the coverage of various dimensions of good health. While other databases focus only on the health dimension, the BANMOP covers other areas that affect health, such as economics, social, environment, disasters, and safety. Furthermore, the BANMOP is a cohort project that emphasizes community engagement and its database is continuously updated. The BANMOP offers long-term prospective data collection. Self-reported health data through web-based or application has strengths and weaknesses. One benefit is that this approach may allow participants to feel more comfortable sharing information on sensitive subjects such as drug use, domestic violence, and sexual activities. At the same time, reporting biases may be present, and measuring self-perceived health status without objective instruments may raise concerns about validity.

## Conclusions

The results of the electronic health databases gathered during the second phase (cross-sectional study) of BANMOP are presented in this first report. The most prevalent NCDs in BANMOP were, hypertension, allergic diseases and diabetes. In addition to context-specific health data, the BANMOP offers health-related data that aids in the development of tailored healthcare interventions and policies for a given community. The BANMOP conceptual framework could be used in other places with environments and contexts comparable to Bangkoknoi. As part of the cohort study, the long-term health data and related data, as well as the results of the projects that were established in phase 3 of BANMOP, will be reported after this initial report. More research should be done on health-related issues like teen pregnancy, low financial literacy, family planning and marriage counseling, and STDs.

## Data Availability

BANMOP population data such as age, gender, incomes are available in a dashboard format at https://www.bangkoknoimodel.com/stat. In-depth data are available in Excel format in Thai and English at https://www.bangkoknoimodel.com/stat. To protect participant privacy, the researcher must submit a request form on the website and wait for approval from the BANMOP committee to access the database.

## Abbreviations

BANMOP: The Bangkoknoi Model Project
MOPH: The Ministry of Public Health
WHO: World Health Organization
UN: The United Nations
SDG: Sustainable Development Goals
NCD: Noncommunicable diseases
NHES: The Thai National Health Examination Survey
GPS: The Global Positioning System
SIRB: The Institutional Review Board of Siriraj Hospital Faculty of Medicine, Mahidol University
ADHD: Attention-deficit/hyperactivity disorder
WMH-CIDI: World Mental Health Composite International Diagnostic Interview
GDP: Gross domestic product
CPR: Cardiopulmonary resuscitation
CSR: Corporate social responsibility
CBR: Community-based rehabilitation)
DSPM: Developmental surveillance and promotion manual
